# Mapping 15 years of crayfish plague in the Iberian Peninsula: The impact of two invasive species on the endangered native crayfish

**DOI:** 10.1371/journal.pone.0219223

**Published:** 2019-08-08

**Authors:** Laura Martín-Torrijos, Harri Kokko, Jenny Makkonen, Japo Jussila, Javier Diéguez-Uribeondo

**Affiliations:** 1 Department of Mycology, Real Jardín Botánico (RJB-CSIC), Madrid, Spain; 2 Department of Environmental and Biological Sciences, University of Eastern Finland, Kuopio, Suomi-Finland; Uppsala Universitet, SWEDEN

## Abstract

Crayfish plague, caused by the pathogen *Aphanomyces astaci*, is one of the main factors responsible for the decimation of the native European crayfish species *Austropotamobius pallipes*. In Spain, two North American freshwater crayfish species, *Procambarus clarkii* and *Pacifastacus leniusculus*, were intentionally introduced during the 1970s for aquaculture and fishery purposes. Since then, incidences of crayfish plague have been continually reported. In this work, we evaluated more than 50 diagnosed cases of crayfish plague that have occurred in the Iberian Peninsula since 2004 by performing a microscopic examination of infected specimens and by molecularly identifying and haplotyping the pathogen. Our results showed that (i) the pathogen *A*. *astaci* has been active 45 years since the first introductions of the invasive North American crayfish species in the Iberian Peninsula, and (ii) *P*. *clarkii* and *P*. *leniusculus* are chronic reservoirs of the crayfish plague pathogen. Moreover, our data confirmed a correspondence between pathogen origin and spread and the specific haplotypes carried by the North American invasive crayfish located in the vicinity of each outbreak. We generated a crayfish plague incidence map of the Iberian Peninsula that shows (i) a northern area, mainly inhabited by alien *P*. *leniusculus*, where crayfish plague cases are associated with the b-haplotype specific to *P*. *leniusculus*, and (ii) southern, central and eastern areas that are basically inhabited by alien *P*. *clarkii*, where crayfish plague cases are associated with the d1- and d2-haplotypes specific to *P*. *clarkii*. The results presented here are evidence of the long standing and negative impact of the two invasive crayfish species on the native species, indicating the need for more extensive control measures.

## Introduction

Biological invasions have increased in magnitude and frequency, particularly due to a rise in global human connectivity [1], and invasive alien species (IAS) are one of the main reasons for local species extinctions [1, 2]. In Europe, more than 12,000 species have been classified as alien, of which 15% are considered IAS [3]. Invasive alien species exert a greater negative effect on aquatic freshwater ecosystems than terrestrial ones [4] and, therefore, constitute one of the main threats to freshwater biodiversity.

Additionally, IAS often act as vectors of pathogens, compounding their effect on ecosystems. Many of the pathogens carried by IAS, such as *Aphanomyces astaci* Schikora, 1906, are especially virulent in new hosts and ecosystems [1], particularly freshwater ones. *Aphanomyces astaci*, which is chronically carried by North American freshwater crayfish species, is responsible for the crayfish plague disease that has decimated native crayfish populations throughout Europe since 1859 [5, 6]. Although *A*. *astaci* and its natural hosts have a balanced relationship as a result of their evolutionary history [7], Australasian, European and South American freshwater crayfishes are more easily infected and more likely to die as a consequence of the infection caused by these virulent form of pathogen [7, 8]. However, this balanced host-pathogen relationship seem to be altered in some naturalized North American crayfish populations in Europe that have become more susceptible to *A*. *astaci* [9–13].

**Fig 1:**
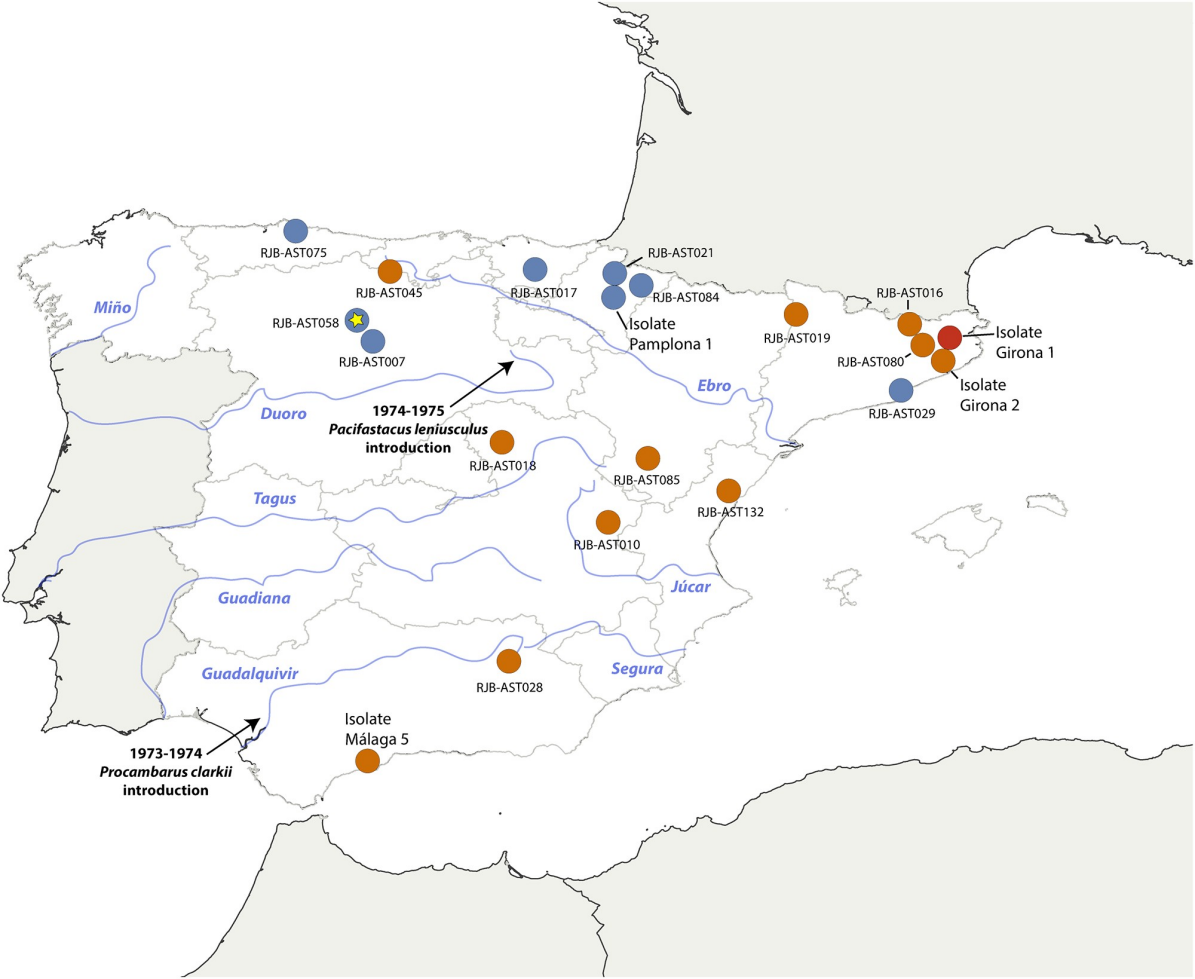
Map showing the location of analyzed crayfish plague outbreaks across the Iberian Peninsula according to mtDNA haplotype. The crayfish plague outbreaks that were haplotyped, including the four isolates, are represented by colored dots and RJB sample numbers: blue dots indicate localities with the b-haplotype (the star indicates an acute case of crayfish plague in a *P*. *leniusculus* population), orange dots indicate localities with the d1-haplotype and the red dot indicates a locality with the d2-haplotype. The geographical location of the principal rivers in Spain appear in blue and the first *P*. *clarkii* and *P*. *leniusculus* introductions are signaled with black arrows.

In Spain, two North American freshwater crayfish, the red swamp crayfish *Procambarus clarkii* Girard, 1852, and the signal crayfish *Pacifastacus leniusculus* Dana, 1852, were intentionally introduced during the 1970s for aquaculture and fishery purposes [14]. *Procambarus clarkii* was first introduced into Guadalquivir marshlands (Fig 1), and surrounding areas, between 1973 and 1974 [15, 16]. Although the species initially occupied areas of central and southern Spain, and also Douro and Ebro river catchments [15, 16], by the end of the 1970s, its popularization had led to its rapid translocation throughout the Iberian Peninsula.

*Pacifastacus leniusculus* was first introduced in Spain between 1974 and 1975 (Fig 1) [15]. The spread of this species followed a different pattern than that of *P*. *clarkii*. Its first introductions took place in hatcheries in the upper catchments of the Douro and Tagus rivers, followed by translocations to nearby areas. In the late 1980s, *P*. *leniusculus* was introduced in northern Spain as a consequence of fishery stocking programs carried out by local administrations in the provinces of Castile and León, Navarre and the Basque Country [14]. In the 1990s, increased demand for crayfishing of *P*. *leniusculus* populations prompted the spread of the species to new catchment areas in central and southern Spain.

Soon after these introductions, the number of populations of the native white-clawed crayfish, *Austropotamobius pallipes* Lereboullet, 1858, started to decrease abruptly as a consequence of the pathogen *A*. *astaci* carried by these IAS [17]. By the end of the 1970s, up to one third of the initial native crayfish populations were estimated to have been lost [14]. In the following decades, the steady decline of the native crayfish was characterized by an estimated trend of regression that ranged up to 50% every five years [14]. Remaining *A*. *pallipes* populations are still critically endangered and survive only in isolated habitats, often thriving in upper catchments that are physically separated from main river basins within their original habitat range [18].

Since 2004, more than 100 crayfish plague outbreaks have been detected and identified in the Iberian Peninsula with the use of microscopy, strain-isolations and molecular analyses with specific diagnostic primers [19, 20]. For instance, the origin of several outbreaks in northern Spain were identified molecularly through the analysis of random amplified polymorphic DNA (RAPD) and amplified fragment length polymorphism (AFLP) markers [21, 22]. These studies showed that *P*. *clarkii* and *P*. *leniusculus* were responsible for outbreaks in northern regions of Burgos and in the Pyrenees, respectively. However, these types of analyses require the isolation of the pathogen in a pure culture, which can be challenging as fresh samples are often difficult to obtain and tissues are usually preserved in 70% ethanol. More recently, a microsatellite method was designed to differentiate genetic groups directly from clinical samples [23]. However, this technique is costly, and potential cross amplifications of other infectious species may lead to misleading results [24].

The reliable and accurate identification of *A*. *astaci* genetic groups requires an efficient analytic tool that provides a means to track the detected pathogen back to its original host. The most common North American freshwater crayfishes, *P*. *clarkii*, *P*. *leniusculus* and *Faxonius limosus* Rafinesque, 1817, chronically carry a specific *A*. *astaci* RAPD group (B-, D- and E, respectively) [21, 25, 26]. These genetic RAPD groups were recently assigned to distinctive haplotypes and hosts using a novel haplotyping method based on mitochondrial ribosomal small (rnnS) and large (rnnL) subunit sequences [27]. This technique has provided accurate data regarding the haplotypes present in a number of clinical samples of crayfish plague [27, 28]. Each haplotype seems to correspond to only one of the North American crayfish species, e.g., the b-haplotype corresponds to *P*. *leniusculus*, and the d1-, d2- and d3-haplotypes correspond to *P*. *clarkii*. Currently, six *A*. *astaci* haplotypes have been identified: a, b, d1, d2, d3 and e. Thus, the haplotyping technique is a tool that can provide key information on the epidemiology, prevalence and virulence of the disease in freshwater ecosystems. Furthermore, the results derived from such studies will allow us to better determine how this pathogen is reaching threatened populations.

In this study, we assessed the impact of *A*. *astaci* strains carried by two rapidly spreading IAS, *P*. *clarkii* and *P*. *leniusculus*, on native *A*. *pallipes* populations in the Iberian Peninsula. Specifically, we used a novel haplotyping technique based on mitochondrial DNA (mtDNA) [27] to molecularly analyze crayfish plague outbreaks detected in the Iberian Peninsula since 2004. We also analyzed the correspondence between the *A*. *astaci* haplotype detected and the distribution of the two IAS carriers.

## Material and methods

### Ethical statement

All experimental procedures and animal manipulations, as well as field sampling, were performed in accordance with current EU and Spanish legislation. All analyses were carried out according to the regulations of the Spanish Ministry of Economy and Competitiveness (MINECO). Under Spanish law, no additional permits or ethical approvals were required for laboratory studies with arthropod invertebrates. Moreover, this study was carried out in strict accordance with the recommendations and protocols established in previous studies.

### Crayfish sampling

A total of 64 *A*. *pallipes* samples collected from crayfish plague events detected since 2004 in Spain were selected from the Crayfish Collection of the Real Jardín Botánico–CSIC (RJB–CSIC), as were 10 samples of either *P*. *clarkii* or *P*. *leniusculus* that had been collected from different populations (Table 1). In this collection, specimens from the same crayfish plague event or IAS population are all stored in the same container. All samples are preserved in 70% ethanol and labeled with the location and date of the outbreak. Selected crayfish specimens were manipulated individually, and tools were sterilized before and after each manipulation to avoid cross contamination between samples.

**Table 1:** Location and collection number of the analyzed crayfish species. Host species of each of the analyzed populations, i.e., *Austropotamobius pallipes*, *Procambarus clarkii* and *Pacifastacus leniusculus* sampled from the Real Jardín Botánico crayfish collection, including corresponding collection codes and year. The location of the host species and the nearest invasive alien species (IAS) is indicated (the (X) indicates IAS were not checked in the surroundings). Result of the microscopy analysis and diagnostic PCR with *A*. *astaci* primers 42 and 640 are also provided: (+) indicates visible signs of disease were observed or the PCR fragment was successfully amplified and the (-) indicates no signs of disease were observed or (--) the PCR was not amplified in the respective analyses. The mitochondrial haplotype of some of the analyzed specimens is also provided (in bold).

Host species	Collection code	Year	Location	Nearest IAS	Microscopy	PCR-42-640	Haplotype
***A*. *pallipes***	**SAP-0471**	**2007**	**Isolate Girona 1/ Isolate AP03 (Crayfish Farm-Olot)**	***P*. *clarkii***	**+**	**+**	**d2**
***A*.*pallipes***	**SAP-XXXX**	**2014**	**Isolate Girona2/Girona 66 (Rio Llemena)**	***P*. *clarkii***	**+**	**+**	**d1**
***A*.*pallipes***	**SAP-0879**	**2009**	**Isolate Málaga 5 (Río Turón)**	***P*. *clarkii***	**+**	**+**	**d1**
***A*.*pallipes***	**SAP-2656**	**2006**	**Isolate Pamplona 1 (Crayfish Farm-Anotz)**	***P*.*leniusculus***	**+**	**+**	**b**
*A*.*pallipes*	RJB-AST001	2011	Granada (Crayfish Farm-Anotz)	*P*. *clarkii*	+	+	--
*A*.*pallipes*	RJB-AST002	2011	Jaen (Arroyo Membrillo)	*P*. *clarkii*	+	+	--
*A*.*pallipes*	RJB-AST003	2012	Granada (Arroyo De La Hermita)	*P*. *clarkii*	+	+	--
*A*.*pallipes*	RJB-AST004	2012	Girona (Muga)	*P*. *clarkii*	+	+	--
*A*.*pallipes*	RJB-AST006	2007	Girona (Muga)	*P*. *clarkii*	+	+	--
***A*.*pallipes***	**RJB-AST007**	**2008**	**Palencia (La Pernia)**	***P*.*leniusculus***	**+**	**+**	**b**
*A*.*pallipes*	RJB-AST008	2009	Girona (Ripolles)	*P*. *clarkii*	+	--	--
*A*.*pallipes*	RJB-AST009	2009	Girona (Valle De Byana)	*P*. *clarkii*	+	--	--
***A*.*pallipes***	**RJB-AST010**	**2009**	**Cuenca (Molinillo)**	**X**	**+**	**+**	**d1**
*A*.*pallipes*	RJB-AST011	2012	Granada (Arroyo De La Hermita)	*P*. *clarkii*	+	+	--
*A*.*pallipes*	RJB-AST012	2005	Alava (Altube)	*P*.*leniusculus*	+	+	--
*A*.*pallipes*	RJB-AST013	2012	Granada (Arroyo De La Hermita)	*P*. *clarkii*	+	+	--
*A*.*pallipes*	RJB-AST014	2005	Girona (Bianya Garrotxa)	*P*. *clarkii*	+	+	--
*A*.*pallipes*	RJB-AST015	2005	Alava (Inoso-Altube)	*P*.*leniusculus*	+	+	--
***A*.*pallipes***	**RJB-AST016**	**2005**	**Girona (Ripolles)**	***P*. *clarkii***	**+**	**+**	**d1**
***A*.*pallipes***	**RJB-AST017**	**2005**	**Alava (Olarte)**	***P*.*leniusculus***	**+**	**+**	**b**
***A*.*pallipes***	**RJB-AST018**	**2004**	**Guadalajara (Tajuña)**	**X**	**+**	**+**	**d1**
***A*.*pallipes***	**RJB-AST019**	**2009**	**Lleida (Crayfish Farm-Pont de Suert)**	***P*. *clarkii***	**+**	**+**	**d1**
*A*.*pallipes*	RJB-AST020	2004	Navarra (Regata Arteki Olagüe)	*P*.*leniusculus*	+	+	--
***A*.*pallipes***	**RJB-AST021**	**2006**	**Navarra (Regata Gambo Lantz)**	***P*.*leniusculus***	**+**	**+**	**b**
*A*.*pallipes*	RJB-AST022	2005	Alava (Inoso Altube)	*P*.*leniusculus*	+	--	--
*A*.*pallipes*	RJB-AST024	2008	Girona (Riera De Joannettes)	*P*. *clarkii*	+	+	--
*A*.*pallipes*	RJB-AST025	2007	Navarra (Cantera De Ofitas)	*P*.*leniusculus*	+	--	--
*A*.*pallipes*	RJB-AST026	2006	Alava (Ulliberri)	*P*.*leniusculus*	+	--	--
*A*.*pallipes*	RJB-AST027	2006	Alava (Balsa De Izarra)	*P*.*leniusculus*	+	+	--
***A*.*pallipes***	**RJB-AST028**	**2011**	**Jaen (Rio Borosa)**	***P*. *clarkii***	**+**	**+**	**d1**
***A*.*pallipes***	**RJB-AST029**	**2011**	**Barcelona (Arro De La Seita Sena De Luna)**	***P*.*leniusculus***	**+**	**+**	**b**
*A*.*pallipes*	RJB-AST030	2011	Navarra (Regata Zaldazain)	*P*.*leniusculus*	+	--	--
*P*.*leniusculus*	RJB-AST031	2007	Burgos (Rio Arlanza Castillo Del Val)	X	-	+	--
*P*.*leniusculus*	RJB-AST032	2007	Navarra (Rio Ultzama Ostiz)	X	-	+	--
*P*.*leniusculus*	RJB-AST033	2008	Navarra (Rio Erro)	X	-	+	--
*A*.*pallipes*	RJB-AST034	2014	Girona (La Arnera)	*P*. *clarkii*	+	--	--
*P*. *clarkii*	RJB-AST038	2011	Jaen (Río Borosa)	X	-	+	--
*A*.*pallipes*	RJB-AST039	2008	Navarra (Etzaburu)	*P*.*leniusculus*	+	+	--
*A*.*pallipes*	RJB-AST040	2008	Navarra (Regata Arteki)	*P*.*leniusculus*	+	--	--
*A*.*pallipes*	RJB-AST041	2008	Navarra (Regata Idozin)	*P*.*leniusculus*	+	--	--
*A*.*pallipes*	RJB-AST042	2008	Navarra (Barranco Sandoain)	*P*.*leniusculus*	+	--	--
*P*.*leniusculus*	RJB-AST043	2008	Navarra (Barranco Tejeria Osteriz)	X	-	--	--
*A*.*pallipes*	RJB-AST044	2008	Navarra (Rio Areta)	*P*.*leniusculus*	+	+	-
***A*.*pallipes***	**RJB-AST045**	**2008**	**Palencia (-)**	**X**	**+**	**+**	**d1**
*A*.*pallipes*	RJB-AST046	2008	Alava (-)	*P*.*leniusculus*	+	+	-
*P*.*leniusculus*	RJB-AST047	2008	Navarra (Regata Izal Salazar)	X	-	--	--
*P*.*leniusculus*	RJB-AST048	2008	Navarra (Regata Elia)	X	-	--	--
*A*.*pallipes*	RJB-AST051	2010	Alava (-)	*P*.*leniusculus*	+	+	--
*A*.*pallipes*	RJB-AST052	2010	Alava (-)	*P*.*leniusculus*	+	+	--
*A*.*pallipes*	RJB-AST053	2010	Jaen (Río Borosa)	*P*. *clarkii*	+	--	--
*A*.*pallipes*	RJB-AST056	2011	Albacete (Río De La Mesta)	*P*. *clarkii*	+	+	-
***P*.*leniusculus***	**RJB-AST058**	**2008**	**Palencia (La Pernia)**	**X**	**+**	**+**	**b**
*A*.*pallipes*	RJB-AST059	2007	Granada (Arroyo De La Hermita)	*P*. *clarkii*	+	+	--
*P*.*leniusculus*	RJB-AST060	2011	Navarra (Rio Ega)	X	-	+	--
*A*.*pallipes*	RJB-AST062	2012	Navarra (Crayfish Farm-Anotz)	*P*.*leniusculus*	+	+	--
*A*.*pallipes*	RJB-AST066	2012	Jaen (Arroyo De La Mesa)	*P*. *clarkii*	+	+	--
*A*.*pallipes*	RJB-AST067	2012	Navarra (Balsa Erna)	*P*.*leniusculus*	+	--	--
*A*.*pallipes*	RJB-AST068	2010	Navarra (Crayfish Farm-Anotz)	*P*.*leniusculus*	+	--	--
*A*.*pallipes*	RJB-AST073	2012	Navarra (Anotz)	*P*.*leniusculus*	+	+	--
*A*.*pallipes*	RJB-AST074	2016	Barcelona (Les Guilleries)	*P*.*leniusculus*	+	+	--
***A*.*pallipes***	**RJB-AST075**	**2015**	**Asturias (-)**	**X**	**+**	**+**	**b**
***A*.*pallipes***	**RJB-AST080**	**2014**	**Girona (Rio Llemena)**	***P*. *clarkii***	**+**	**+**	**d1**
*A*.*pallipes*	RJB-AST081	2012	Jaen (Río Borosa)	*P*. *clarkii*	+	--	--
*A*.*pallipes*	RJB-AST082	2007	Guadalajara (Tajuña)	X	+	--	--
*P*.*leniusculus*	RJB-AST083	2016	Barcelona (Les Guilleries)	X	-	+	--
***A*.*pallipes***	**RJB-AST084**	**2015**	**Navarra (Artanga)**	***P*.*leniusculus***	**+**	**+**	**b**
***A*.*pallipes***	**RJB-AST085**	**2017**	**Teruel (Arroyo Valtablao)**	***P*. *clarkii***	**+**	**+**	**d1**
*A*.*pallipes*	RJB-AST121	2015	Girona (Lésquirol)	*P*. *clarkii*	+	--	--
*A*.*pallipes*	RJB-AST122	2015	Tarragona (Capafonts)	*P*. *clarkii*	+	+	--
*A*.*pallipes*	RJB-AST124	2015	Guipuzcua (Río Urola)	*P*.*leniusculus*	+	--	--
*A*.*pallipes*	RJB-AST125	2015	Huesca (Barranco De Villano)	X	+	--	--
*A*.*pallipes*	RJB-AST126	2015	Segovia (Rio Eresma)	X	+	--	--
*A*.*pallipes*	RJB-AST128	2015	Palencia (-)	X	+	--	--
*A*.*pallipes*	RJB-AST129	2015	Girona (Olot)	*P*. *clarkii*	+	--	--
*A*.*pallipes*	RJB-AST131	2016	Girona (Camdoca)	X	+	--	--
***A*.*pallipes***	**RJB-AST132**	**2017**	**Castellón (Pobla De Benifassá)**	***P*. *clarkii***	+	**+**	**d1**
*A*.*pallipes*	RJB-AST133	2017	Teruel (Río Pena)	*P*. *clarkii*	+	--	--
*A*.*pallipes*	RJB-AST134	2017	Teruel (Río Blanco)	*P*. *clarkii*	+	--	--

### Microscopic examination of infected crayfish and the molecular identification of the *A*. *astaci* pathogen

For the microscopic analyses, the subabdominal cuticles were carefully removed and handled individually due to the fragility of the older samples. Cuticles were observed for visual signs of the presence of the pathogen, specifically non-melanized or melanized hyphae, on an inverted Olympus CKX41SF microscope (Olympus Optical, Tokyo, Japan).

For the molecular analyses, various tissues were collected from each individual including a subabdominal cuticle, the walking legs and a fragment of the telson and the joint of the chelae, and stored in 70% ethanol until further processing. Prior to DNA isolation, the tissue samples were rehydrated in TE buffer (TRIS 10 mM/ EDTA 1 mM, pH 8) by rinsing each sample up to three times with TE and then leaving it overnight in the buffer. Samples were transferred into 2-ml tubes that were previously frozen at -80 °C prior to being lyophilized in a VirTis BenchTop K freeze dryer for 24 hours (≤-50 °C; ≤ 20 mTorr). Samples were subsequently homogenized using a TissueLyser (Qiagen, Germany). The E.Z.N.A. Insect DNA Kit (Omega Bio-tek, Norcross, Atlanta, USA) was used to isolate genomic DNA. To molecularly test for the presence of the *A*. *astaci* pathogen, a fragment of the internal transcribed spacer (ITS) region was amplified using the diagnostic primers 42 [19] and 640 [20] (which amplify ITS1, the 5.8S rDNA and ITS2) in a single round of PCR according to the assay described by Oidtmann et al. 2006 [19]. DNA extracted from a pure culture of *A*. *astaci* strain AP03, whose genome has been sequenced, was used as the positive control [29]; distilled Milli-Q water was used as the negative control. Amplified products (3 μL of each reaction) were analyzed by electrophoresis in 1% agarose TAE gels stained with SYBR Safe (Thermo Fisher Scientific, Waltham, Massachusetts, USA). Sequencing of both strands of positive products was performed using an automated sequencer (Applied Biosystems 3730xl DNA Analyzer, Macrogen, Netherlands). Sequences were aligned and edited using the program Geneious 10.0.2 [30]. A BLAST search was performed to verify the identity of each sequence.

### Sequencing, phylogenetics and haplotyping

To characterize the phylogenetic relationships and haplotypes of the *A*. *astaci* isolates, the mitochondrial ribosomal small (rnnS) and large (rnnL) subunits were amplified using the primer pairs AphSSUF/AphSSUR and AphLSUF/AphLSUR, respectively, as described by Makkonen et al. 2018 [27]. Genomic DNA that tested positive for the presence of *A*. *astaci* with diagnostic primers 42 [19] and 640 [20] were used as templates in these amplifications. Four *A*. *astaci* single spore isolates, Isolate Málaga 5 (SAP-0879), Isolate Pamplona 1 (SAP-2656), Isolate Girona 1/AP03 (SAP-0471) and Isolate Girona 2/Girona 66 (SAP-XXXX), were also sequenced and included in our phylogenetic analyses. Positive and negative controls (*A*. *astaci* strain AP03 [29] and distilled Milli-Q water, respectively) were included. Amplified products were analyzed by electrophoresis as described above and then purified using a QIAquick PCR Purification Kit (Qiagen, Hilden, Germany). Sequencing and sequence alignment and editing were performed as described above.

Phylogenetic approximations based on Bayesian inference (BI) and maximum likelihood (ML) were used to reconstruct relationships. The BI analysis was performed in MrBayes v.3.2.6 [31] using the MCMC method with 10 million generations, three runs (8 chains per run) with a burn-in of 25% and a standard deviation of split frequencies <0.01. Nodes with posterior probability (pp) values ≥0.95 were considered supported. The ML analysis was performed using RAxML v.8, [32] as implemented in raxmlGUI v1.5b1 [33], with 100 independent replicates and 1000 rapid bootstraps. Nodes with bootstrap values ≥75 were considered supported. The resulting trees from the BI and ML analyses were visualized with FigTree v1.4.2 [34]. Sequences of the mtDNA regions rnnS and rnnL of isolates analyzed in previous studies [27, 28], available in GenBank were also included in our analyses. *Aphanomyces frigidophilus* was used as the outgroup in both phylogenetic approximations. Analyses were performed with rnnS and rnnL individually, as well as with a concatenated rnnS and rnnL dataset, using the same parameters described above.

Mutational changes between sequences in the most parsimonious haplotype network were estimated using TCS v.1.21 [35], and the genealogical relationships were visualized with PopArt v1.7.2 [36].

### Correspondence of *A*. *astaci* haplotypes associated with crayfish plague outbreaks in the Iberian Peninsula with the nearest IAS

For most of the crayfish plague outbreak events, a putative nearest IAS was assigned based on information provided by the local conservation authorities monitoring crayfish populations in each province (Table 1). Haplotyping data obtained in our analyses were used to corroborate this information and to generate a crayfish plague map of the Iberian Peninsula.

## Results

### Microscopic examination of infected crayfish and the molecular identification of the *A*. *astaci* pathogen

Microscopic examination of the subabdominal cuticles revealed the presence of abundant growth of non-melanized hyphae on all 64 *A*. *pallipes* samples (Table 1). The growth was characteristic of an *A*. *astaci* infection: round hyphal tips, all having approximately the same diameter (ca 10 μm). Melanized hyphae, characteristic of chronic infections, were not observed on the *P*. *clarkii* and *P*. *leniusculus* samples, except for *P*. *leniusculus* sample RJB-AST058 (Table 1), which presented a few melanized but abundant non-melanized hyphae, characteristic of an acute crayfish plague infection [37].

Using the diagnostic primers described above [19, 20], the presence of *A*. *astaci* was confirmed molecularly in 40 of the 64 crayfish plague cases. A GenBank BLAST search of the obtained sequences showed 100% similarity to *A*. *astaci* isolates SAP0877 (accession number KX555484), AC14-025 (accession number KU159681), PL_Ve1-T42m (accession number JX272193) and SAP302 (accession number FM999249).

### Sequencing, phylogenetics and haplotyping

**Fig 2:**
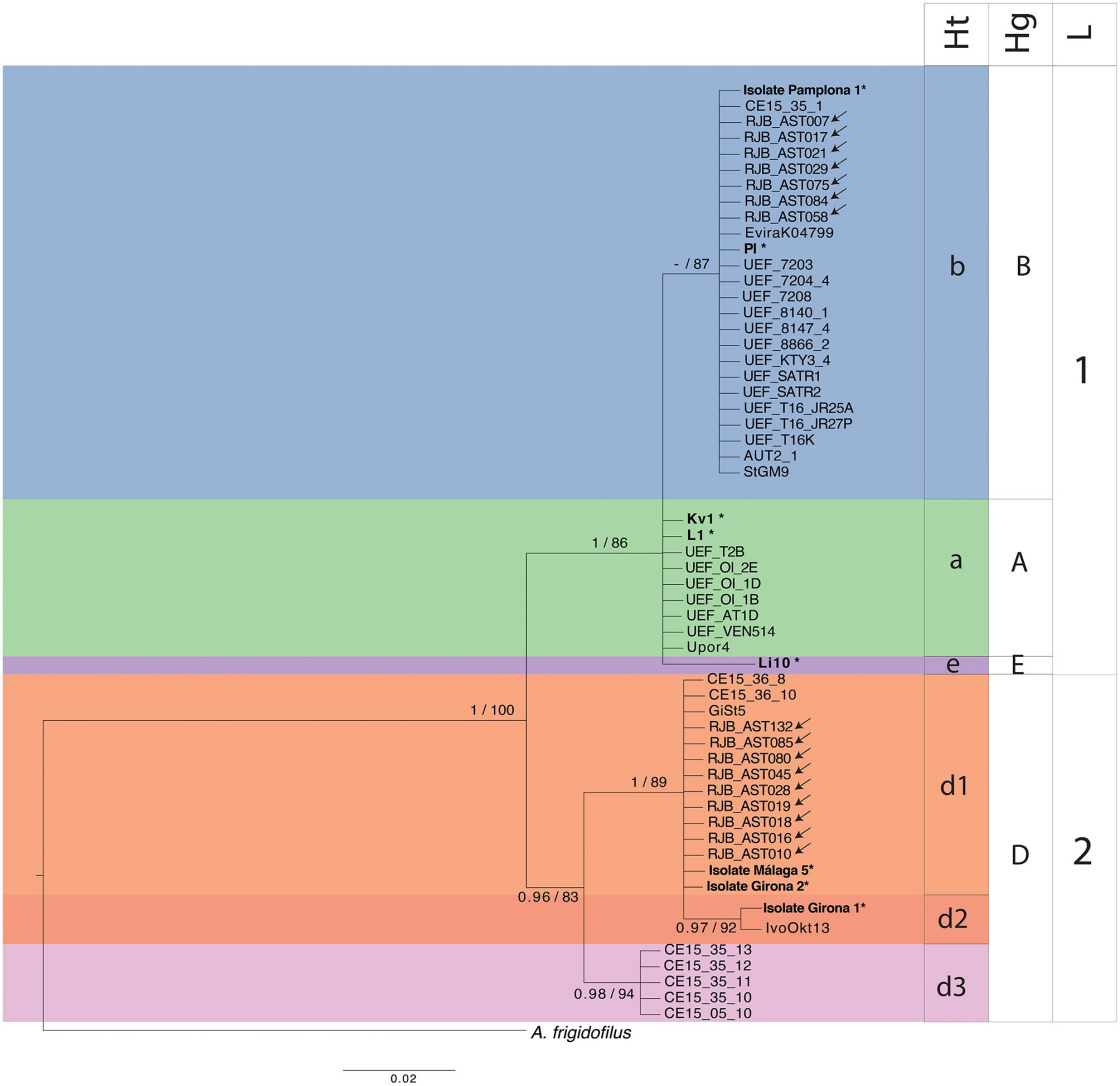
Phylogenetic analyses of *A*. *astaci* mitochondrial regions. Bayesian inference analyses based on concatenated dataset of *A*. *astaci* rnnS + rnnL sequences obtained from clinical samples originating from the native crayfish *Austropotamobius pallipes* (arrows), or the invasive North American crayfishes *Procambarus clarkii* (arrows) and *Pacifastacus leniusculus* (arrows), that were collected in the Iberian Peninsula and housed in the RJB Crayfish Collection. Values above branches represent Bayesian posterior probabilities (>0.95) and maximum likelihood bootstrap supports (>75), respectively. Scales bar for phylogenetic analysis indicates substitutions per site. The original strains used as references and identified in previous studies by RAPD-PCR [14, 22, 23] are indicated in bold and with an asterisk (*), and correspond to RAPD group A: L1*, RAPD group B: Pl*, RAPD group C: Kv1*, RAPD group D: AP03* and RAPD group E: Li10*. Abbreviations: Ht, haplotypes; Hp, haplogroups and L, lineages.

Amplification of mitochondrial rnnS and rnnL fragments was successful for 16 of the 40 *A*. *astaci*-positive samples, corresponding to 15 cases of crayfish plague in *A*. *pallipes* and one mass mortality event in a *P*. *leniusculus* population. Mitochondrial rnnS and rnnL sequences were also successful obtained for the four *A*. *astaci* single spore isolates. Amplicon sizes for rnnS and rnnL were 475 base-pairs (bp) and 355 bp, respectively (GenBank accession number for rnnS MK872961-MK872980 and for rnnL MK872981-MK873000). Similar and congruent topologies were recovered in the BI and ML independent analyses of rnnS and rnnL (S1 Fig). Analyses revealed that the sequences from the diagnosed crayfish plague events correspond to two different haplotypes and haplogroups: the b-haplotype within the B-haplogroup and the d1-haplotype within the D-haplogroup (Table 1, Fig 2). The four *A*. *astaci* isolates correspond to three different haplotypes within two haplogroups: the b-haplotype within the B-haplogroup and the d1- and d2-haplotypes within the D-haplogroup.

The haplotype diversity found in the rnnS and rnnL regions supports the results of the phylogenetic analyses (S1 Fig). The concatenated rnnS and rnnL dataset showed a total of 11 segregating sites, confirming the presence of three of the six known haplotypes (S1 Fig).

### Correspondence of *A*. *astaci* haplotypes associated with crayfish plague outbreaks in the Iberian Peninsula with the nearest IAS

Mapping of the mtDNA haplotypes obtained in our analyses validates the information provided by local authorities on the proximity of the closest IAS to the different crayfish plague events (Fig 1, Table 1). Crayfish plague events RJB-AST007, 017, 021, 029, 058, and 084, all designated with the b-haplotype, occurred in localities where *P*. *leniusculus* is known in the vicinity. Crayfish plague events RJB-AST016, 019, 028, 080, 085 and 132, all designated with the d1-haplotype, occurred in localities where *P*. *clarkii* is known in the vicinity. Moreover, in four of the cases without an assigned nearest IAS (i.e., RJB-AST010,018, 045 and 075), the haplotype found suggests which of the two invasive species was responsible for spreading the pathogen.

## Discussion

The decimation of the Iberian crayfish populations started in the 1970s and continues to the present day. This decline represents one of the most extreme examples of the biodiversity crisis ever described for freshwater ecosystems [38]. It has been estimated that more than 80% of the native populations in the Iberian Peninsula have disappeared [14]. As a result of this emergent pathogen, annual crayfish industry catches of *A*. *pallipes* in Spain dropped from about 2,000 tons/year in 1972 to zero in less than a decade [14]. Since the introduction of this pathogen through the invasive North American crayfish species, native crayfish populations have been in continuous decline. Meanwhile, these IAS have rapidly naturalized and spread throughout the Iberian Peninsula, the impact of which has not yet been evaluated for the region as a whole, in spite of the high extinction risk of the *A*. *pallipes* in most regions of Spain and the near extinction of this species in Portugal [14, 39].

Monitoring the spread of the crayfish plague and accurately estimating the impact of the causative pathogen is difficult, particularly given the limited financial resources of the regional governments. Moreover, the scarce remaining *A*. *pallipes* populations in the Iberian Peninsula only thrive in difficult to access mountainous regions and upstream brooks. This and the fact that outbreaks occur rapidly, often leaving no visible signs of the disease, further complicate the detection of crayfish plague outbreaks among native populations.

Analyses of past crayfish plague outbreaks from diverse locations in Spain has been made possible due to the establishment of a crayfish collection at the RJB–CSIC, and its maintenance of samples from outbreaks since 2004. In this study, we analyzed 64 samples collected from such events reported in Spain over the last 15 years. The results of our analyses show that, 45 years after the introductions of the invasive North American crayfish species, the pathogen *A*. *astaci* is still actively affecting the endangered *A*. *pallipes* in Spain. Although other factors, including drought, poaching, channelization, habitat destruction and water pollution [18], have been cited as responsible for the decline of the *A*. *pallipes*, our data emphasize the constant impact of this disease on native populations. Our data also support previous studies that have shown the damaging effect of *A*. *astaci* from *P*. *clarkii* and *P*. *leniusculus* hosts on *A*. *pallipes* populations, particularly in the Pyrenees. For instance, in the western Pyrenees, it was estimated that *P*. *leniusculus* was responsible for at least 70% decline of *A*. *pallipes* in Navarre within five years [40]. In the eastern Pyrenees, where *P*. *clarkii* is responsible for the main crayfish plague outbreaks among native *A*. *pallipes* populations, a similar level of decline has been reported [22].

Populations of either *P*. *clarkii* or *P*. *leniusculus* are known from local authorities to be present in the near vicinity of most of the analyzed *A*. *pallipes* populations. By analyzing two mitochondrial DNA regions of *A*. *astaci* in clinical samples, we have been able to detect the presence of the pathogen, identify its haplotype and confirm a correlation between pathogen origin and the specific haplotypes of the invasive North American crayfishes located in the vicinity of each outbreak. The *A*. *astaci* b-haplotype, specific to *P*. *leniusculus*, that was sequenced from *A*. *pallipes* samples is identical to the b-haplotypes identified in other regions of the world [27, 28] and also to those obtained from clinical samples of *P*. *leniusculus*, e.g., RJB-AST058, sequenced in this study, which constitutes the first detected crayfish plague outbreak on a *P*. *leniusculus* population in Spain. On the other hand, the alteration of the balance relationship between North American *P*. *leniusculus* and *A*. *astaci* have been reported among populations in Fennoscandia [9–13]. In addition, in our analyses, we detected haplotypes d1 and d2, two of the three known *P*. *clarkii* specific haplotypes [28]. Of the crayfish plague events characterized by d-haplotypes, only one corresponds to the d2-haplotype (Isolate Girona 1); all others correspond to the d1-haplotype. On the other hand, for those 40 *A*. *astaci* cases determined with the diagnostic primers, 25 were not suitable for haplotype characterization due to samples low agent levels. Thus, diversity and the distribution of *A*. *astaci* haplotypes are still unknown, therefore further studies based on the analysis of rnnS and rnnL regions will certainly provide a better understanding of the spread of the haplotypes and their host.

We generated a haplotype specific map of crayfish plague epidemics in Spain which distinguishes two main areas: (i) a northern area, where *P*. *leniusculus* is mainly distributed and where crayfish plague outbreaks in *A*. *pallipes* are associated with its specific b-haplotype, and (ii) an area covering southern, central and eastern regions of Spain, where *P*. *clarkii* is mainly found and where crayfish plague outbreaks are associated with its specific d1- and d2-haplotypes.

The North American crayfish species seem to carry a specific haplotype that is associated with different environmental requirements. For instance, *A*. *astaci* haplotypes specific to *P*. *clarkii* are better adapted to warmer environments than those of P. *leniusculus* [21, 22], adding to the great concern for the conservation of the *A*. *pallipes*, especially under a global warming scenario. Our results confirm once more *P*. *clarkii* and *P*. *leniusculus* as chronic reservoirs of the crayfish plague pathogen that are able to transmit *A*. *astaci* and infect remaining *A*. *pallipes* populations. Wildlife trade and fishery activities have favored the translocations of these chronic carrier species, resulting in an increase in their distribution and thus in the persistence of the pathogen within Iberian freshwater ecosystems over the last 45 years.

*Aphanomyces astaci* is listed among the 100’s world worst invasive species [41]. Five of its chronic carriers hosts, *F*. *limosus*, *Faxonius virilis* Hagen, 1870, *P*. *leniusculus*, *P*. *clarkii* and *Procambarus virginalis* Lyko, 2017, all of which were introduced to Europe, are included in the European Union (EU) list of Invasive Alien Species of Union concern, which currently consists of 37 species [42]. Therefore, conservation strategies that aim to protect *A*. *pallipes* populations should also address the prevention of IAS introductions and translocations following Spanish legislation [43] and EU regulations [42, 44]. Furthermore, environmental programs whose main focus is to protect the native freshwater crayfish, and to identify and control IAS, should be implemented to help in the conservation of this highly endangered freshwater crustacean species.

## Supporting information

S1 Fig Phylogenetic analyses of *A. astaci* mitochondrial regions. Bayesian inference analyses based on *A*. *astaci* rnnS, rnnL, and concatenated rnnS + rnnL sequences obtained from clinical samples originating from the native crayfish *Austropotamobius pallipes* (arrows), or the invasive North American crayfishes *Procambarus clarkii* (arrows) and *Pacifastacus leniusculus* (arrows), that were collected in the Iberian Peninsula and housed in the RJB Crayfish Collection. (**A)** Bayesian inference analysis based on rnnS sequences. (**B**) Bayesian inference analysis based on rnnL sequences. (**C)** Bayesian inference analysis based on concatenated rnnS + rnnL sequences. Values above branches represent Bayesian posterior probabilities (>0.95) and maximum likelihood bootstrap supports (> 75), respectively. Scales bar for phylogenetic analysis indicates substitutions per site. Abbreviations: Ht, haplotypes; Hp, haplogroups and L, lineages.
(TIF)
